# Morpho-histological development of the somatic embryos of *Typha domingensis*

**DOI:** 10.7717/peerj.5952

**Published:** 2018-11-23

**Authors:** Guadalupe Hernández-Piedra, Violeta Ruiz-Carrera, Alberto J. Sánchez, Arlette Hernández-Franyutti, Alfonso Azpeitia-Morales

**Affiliations:** 1 Programa de Maestría en Ciencias Ambientales, Universidad Juárez Autónoma de Tabasco, Villahermosa, Tabasco, México; 2 Universidad Juárez Autónoma de Tabasco, Diagnóstico y Manejo de Humedales Tropicales, Villahermosa, Tabasco, México; 3 Universidad Juárez Autónoma de Tabasco, Biología y Manejo de Organismos Acuáticos, Villahermosa, Tabasco, México; 4 Campo Experimental Huimanguillo, Instituto Nacional de Investigaciones Forestales, Agrícolas y Pecuarias, Tabasco, México

**Keywords:** Sustainable propagation, Embryogenic maturation, Somatic embryogenesis, Histodifferentiation, Cattails, Emergent aquatic macrophyte

## Abstract

**Background:**

Sustainable methods of propagation of *Typha domingensis* through somatic embryogenesis can help mitigate its current condition of ecological marginalization and overexploitation. This study examined whether differentiation up to coleoptilar embryos could be obtained in an embryogenic line proliferated with light and high auxin concentration.

**Methods:**

Murashige and Skoog medium at half ionic strength and containing 3% sucrose and 0.1% ascorbic acid was used for the three embryogenic phases. Induction started with aseptic 9-day-old germinated seeds cultured in 0.5 mg L^−1^ 2,4-dichlorophenoxyacetic (2,4-D). Proliferation of the embryogenic callus was evaluated at 2,4-D concentrations ranging from 0 to 2 mg L^−1^ in cultures maintained in the dark. The dominant embryogenic products obtained in each treatment were used as embryogenic lines in the third phase. Thus, maturation of the somatic embryos (SEs) was analyzed using four embryogenic lines and under light vs. dark conditions. Embryogenic differentiation was also monitored histologically.

**Results:**

Proliferation of the nine morphogenetic products was greater in the presence of 2,4-D, regardless of the concentration, than in the absence of auxin. Among the products, a yellow callus was invariably associated with the presence of an oblong SE and suspended cells in the 2,4-D treatments, and a brown callus with scutellar somatic embryos (scSEs) in the treatment without 2,4-D. During the maturation phase, especially the embryogenic line but also the light condition resulted in significant differences, with the highest averages of the nine morphogenetic products obtained under light conditions and the maximum concentration of auxin (YC3 embryogenic line). Only this line achieved scSE growth, under both light and dark conditions. Structurally complete coleoptilar somatic embryos (colSEs) could be anatomically confirmed only during the maturation phase.

**Discussion:**

In the embryogenic line cultured with the highest auxin concentration, light exposure favored the transdifferentiation from embryogenic callus to scSE or colSE, although growth was asynchronous with respect to the three embryogenic phases. The differentiation and cellular organization of the embryos were compatible with all stages of embryogenic development in other monocotyledons. The growth of colSEs under light conditions in the YC3 embryogenic line and the structurally complete anatomic description of colSEs demonstrated that differentiation up to coleoptilar embryos could be obtained. The diversity of embryogenic products obtained in the YC3 embryogenic line opens up the opportunity to synchronize histological descriptions with the molecules associated with the somatic embryogenesis of *Typha* spp.

## Introduction

Anthropogenic impacts on wetlands threaten environmental processes and services related to native aquatic vegetation. The emergent rooted macrophyte *Typha domingensis* Pers. (southern cattail) is a frequent component of the herbaceous associations that dominate the wetlands of Central and North America (Reddy, Rasineni & Raghavendra, 2010). Like other cattails, *T. domingensis* is an invasive species in freshwater wetlands and its biochemical interactions show ample plasticity, resulting in competitive advantages in widely variable environments (Harrison et al., 2017). Nonetheless, this emergent rooted macrophyte is of environmental value because it sequesters and stores carbon from the atmosphere, provides critical habitats that sustain high biodiversity, and purifies eutrophic and polluted waters (Thorp, Thoms & Delong, 2006; Mitsch et al., 2013; Harrison et al., 2017). However, its populations are threatened by fragmentation, land-use changes, control measures in agricultural practices, and exploitation as a raw material in the production of biofuel (Thorp, Thoms & Delong, 2006; Erwin, 2009; Liu et al., 2012; Mora-Olivo, Villaseñor & Martínez, 2013; Xu et al., 2013; Palomeque-De La Cruz et al., 2017). These pressures justify the development of a sustainable means of *T. domingensis* production to preserve its environmental services and benefits (Liu et al., 2012; He et al., 2015), such as a method of propagation that enables *T. domingensis* extraction independent from the natural environment. For aquatic monocotyledons, with the exception of rice (Indoliya et al., 2016), in vitro methods of asexual or somatic embryogenesis to conserve and propagate germplasm and to achieve the sustainable production of genetic varieties (Von Arnold et al., 2002; Sánchez-Chiang & Jiménez, 2010; Reed et al., 2011) have rarely been applied. Investigations of somatic embryos (SEs) have contributed greatly to understanding the physiological, biochemical, and molecular mechanisms underlying sexual embryogenesis. Such studies have revealed that during SE growth from somatic cells (haploid or diploid) to embryos, morphological changes occur that are similar to those in zygotic embryos (Quiroz-Figueroa et al., 2006; Smertenko & Bozhkov, 2014; Mahdavi-Darvari, Noor & Ismanizan, 2015). Thus, the cultures produce a variety of embryogenic lines that reflect the developmental biology of the SE and its embryogenic pathway (Von Arnold et al., 2002; Wickramasuriya & Dunwell, 2015). The different lines obtained have been explained by the asynchronous nature of the complex process of embryonic transdifferentiation, since somaclonal variation can derive from indirect somatic or cyclic embryogenesis (Horstman, Bemer & Boutilier, 2017) and by genotypic differences in sequential development during somatic embryogenesis (George, Hall & Klerk, 2008). An understanding of the differences resulting from the methods used to produce SEs has been facilitated by histological studies (Máthé et al., 2000; Burris et al., 2009; Vega et al., 2009).

Somatic embryogenesis is a multiphase process that occurs in in vitro cultures and implies the previous installation of a cellular capacity to respond to external molecular signals (Von Arnold et al., 2002). During the inductive phase, signal activation by auxins causes cellular reprogramming toward embryogenic differentiation (Elhiti, Stasolla & Wang, 2013; Fehér, 2015). For example, embryogenic induction and proliferation have been stimulated in monocotyledons by exposure to the auxin 2,4-dichlorophenoxyacetic (2,4-D) (Máthé et al., 2000; Rogers, 2003; Oh et al., 2008). Analogous to the development of zygotic embryos in nature (Von Aderkas et al., 2015), the elimination of auxin exposure during periods of darkness has been frequently applied to obtain competent embryos (Filonova, Bozhkov & Von Arnold, 2000; Guillou et al., 2018). Consequently, experimental studies aimed at quantifying the effect of light on SE maturation have been unable to resolve the possible discrepancies arising from its effects (Devi, Sharma & Ahuja, 2014; Mikuła et al., 2015), because in some ferns, angiosperms, and gymnosperms, light exposure improves SE formation and maturation, both anatomically and biochemically (Mikuła et al., 2015; Von Aderkas et al., 2015; Klubicová et al., 2017). An especially prominent effect of light was demonstrated in the embryonal root cap in a *Larix* × *marschlinsii* hybrid line obtained via secondary embryogenesis (Von Aderkas et al., 2015). These observations raise the question whether differentiation up to coleoptilar embryos can be improved by light manipulation of embryogenic lines grown under very high auxin concentrations. This study examined the morpho-histological development leading to the maturation of *T. domingensis* SEs grown under light and dark conditions in embryogenic lines exposed to a 2,4-D gradient.

## Materials and Methods

### Preparation of the germinated seeds

Mature *T. domingensis* seeds were collected in the catchment area of the Grijalva River, encompassing the city of Villahermosa, Mexico (17°59′N, 92°57′W), located in the basin of the Grijalva and Usumacinta Rivers. Seeds with no perianth were obtained following the method of Lorenzen et al. (2000) and were sterilized first in 30% (v/v) ethanol for 10 min and then with 10% (v/v) bleach (Cloralex, Mexico City, Mexico) for 10 min. After three rinses in type 2 pure sterile water the seeds were cultured under aseptic conditions in sterile culture units at a ratio of 1:50 g mL^−1^ purified water. The culture units, consisting of glass flasks (five cm tall, Ø seven cm) with a Magenta^®^ polycarbonate lid, containing aqueous medium were autoclaved before use at 121 °C and 104 kPa for 25 min in a sterilizer (SM300; Yamato Scientific, Tokyo, Japan). The germinated seeds were grown in a controlled environment at 28 ± 2 °C under cool white light (Philips, USA) at a photon flux density of 20 μmol m^−2^ s^−1^ (Quantum Light Meter; Spectrum Technologies, Illinois, USA) and a photoperiod of 16 h light: 8 h dark.

### Embryogenesis evaluation

The production of SEs occurred in three phases: induction, proliferation of the embryogenic callus, and embryo maturation (Von Arnold et al., 2002; Saenz et al., 2006). The embryos were cultured with shaking under the same conditions of temperature, photon flux density, and photoperiod used for germination, except that during the induction and proliferation phases the cultures remained in darkness. The culture time per phase was 28 days. To satisfy the objectives of each somatic embryogenesis phase, the macroscopic products (as described in the Morphogenetic responses section) were transferred to fresh medium using 6″ straight round-tip tweezers in a laminar flow hood (Veco, Mexico City, Mexico).

The culture medium was prepared with a mixture of Murashige and Skoog basic salts (MS; Murashige & Skoog, 1962) at half ionic strength (MS_0.5_), MS vitamins, 3% sucrose, and 10 mg L^−1^ ascorbic acid as an antioxidant. All medium components were from Sigma–Aldrich (St. Louis, MO, USA). The culture medium was sterilized under the same conditions as for seed germination.

The embryogenic induction phase started with three 9-day-old aseptic germinated seeds cultured in the dark in 0.5 mg L^−1^ 2,4-D (*n* = 48). The next two phases, proliferation and maturation, were induced in two independent experiments of conventional design.

Embryogenic callus proliferation was evaluated based on the presence of morphogenetic features resulting from four treatments (0, 0.5, 1, and 2 mg L^−1^ 2,4-D; *n* = 12 each) in cultures maintained in the dark. In this preliminary experiment, the highest proliferation rate was expected from calli exposed to the highest auxin concentration. The dominant morphogenetic products obtained in each of the four treatments were identified based on their color and the respective embryogenic lines were used in the follow-up experiments on embryonic maturation and transdifferentiation of the SEs up to the coleoptilar stage in the absence of phytoregulator application. Maturation of the four embryogenic lines was evaluated with respect to the effects of light and dark conditions (*n* = 40 = 4 embryogenic lines × 2 light conditions × 5 replicates), whereas transdifferentiation up to the coleoptilar stage was expected to occur only in the presence of light.

### Morphogenetic responses

The absence and presence in the cultures of the morphogenetic products characteristic of the three embryogenic phases, as described in Dodeman, Ducreux & Kreis (1997); Fehér, Pasternak & Dudits (2003); Von Arnold et al. (2002), and Quiroz-Figueroa et al. (2006), were evaluated. These products, which either adhered to the explant or were suspended in the culture medium, were identified during weekly observations using a Zeiss Stemi DV4 stereomicroscope (Zeiss, Göttingen, Germany).

### Histological analysis

The morphogenetic products representative of each treatment and phase were collected from 30% of the culture units and preserved in a formaldehyde–acetic acid–ethanol solution for 24 h. They were then dehydrated in a graded (70–100%) ethanol series (30 min per step), clarified for 1 h with 1:1 ethanol: xylol and 100% xylol (Filonova, Bozhkov & Von Arnold, 2000), and embedded in xylol: paraffin (Paraplast^®^; Sigma–Aldrich, St. Louis, MO, USA) using a Reichert-Jung Mod 8044 automatic tissue embedding device (Cambridge Instruments GmbH, Buffalo, NY, USA). Thick (six μm) serial cross-sections were prepared using a Reichert-Jung Mod. Hn 40 sliding microtome (Cambridge Instruments GmbH, Germany, Buffalo, USA) and stained with toluidine blue and hematoxylin and eosin, both at a concentration of 0.2%. The histological preparations were analyzed using a Zeiss Axiostar Plus light microscope (Carl Zeiss, Göttingen, Germany) equipped with a Zeiss Axiocam digital camera (model MRc5; Carl Zeiss, Göttingen, Germany). The histological analysis of the embryogenic products was qualitative and based on the detection of the cellular and tissue markers described for other monocotyledon species by Máthé et al. (2000), Meneses et al. (2005), Burris et al. (2009), and Vega et al. (2009).

### Statistical analysis

The average percentage of the embryogenic products that developed was analyzed with analysis of variance (ANOVA) or the nonparametric Kruskal–Wallis and Mann–Whitney *U* tests. Single-factor ANOVA was used to analyze the proliferation phase, and two-factor ANOVA the maturation phase. Both were followed by Fisher’s least significant difference post-hoc test. Nonparametric tests were performed for data that did not show a normal distribution (Kolmogorov–Smirnov; *p* < 0.05) or homogeneity of variance (Cochran; *p* < 0.05). All analyses were performed using the Statistica program version 8 (StatSoft, Tulsa, OK, USA).

## Results

### Embryogenic induction

**Figure 1:**
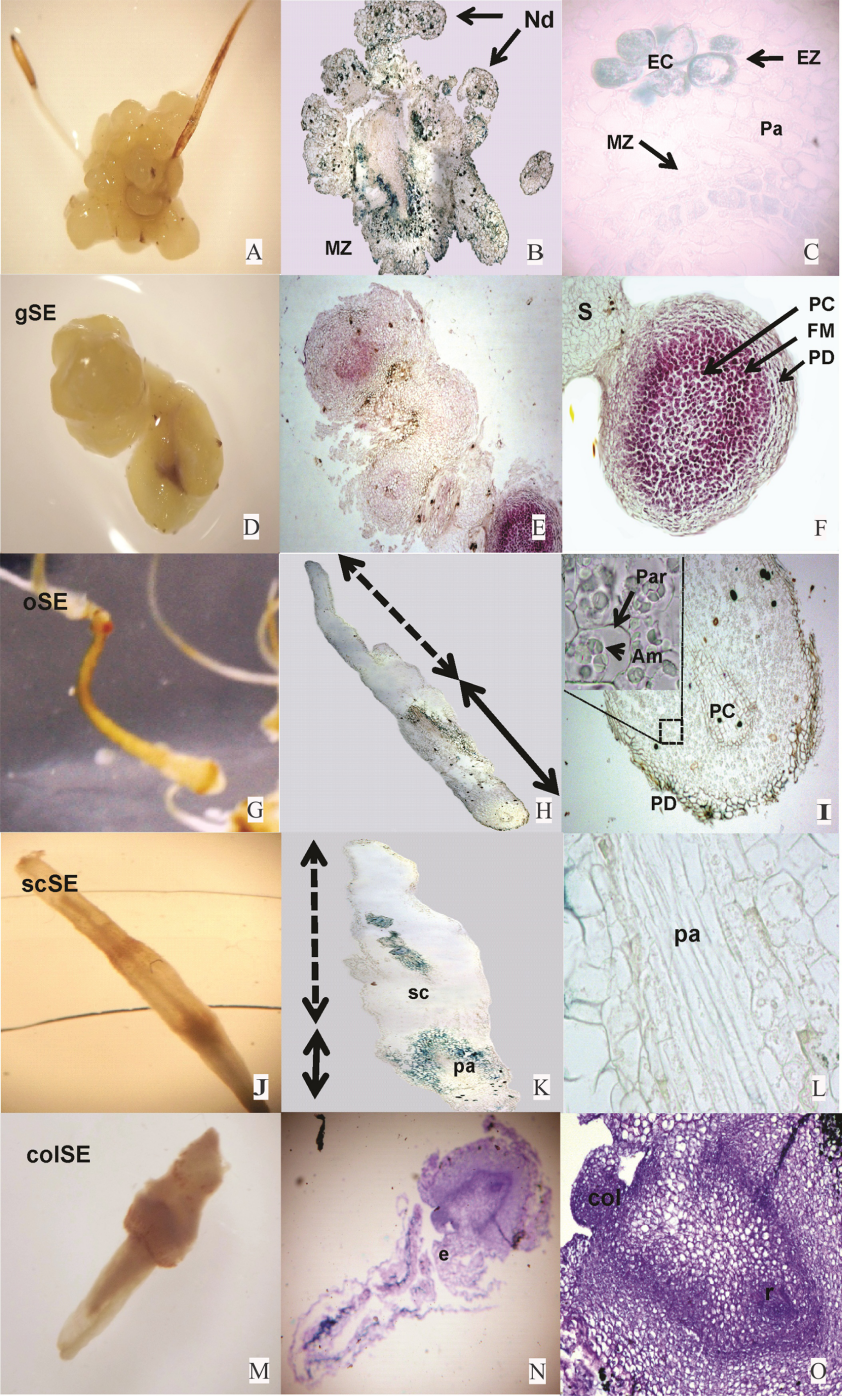
Embryogenic differentiation of *Typha domingensis*. Yellow callus: (A) morphology (8×), (B) cross-section (toluidine blue, 200×), (C) meristematic and embryogenic region (toluidine blue, 400×); globular somatic embryos (gSEs), (D) over-yellowed callus, (E) cross-section (hematoxylin and eosin, 200×), (F) radial pattern formed by three meristems: protoderm, fundamental, and procambium; oblong somatic embryos (oSEs), (G) over-yellowed callus at day 56, (H) longitudinal section showing the suspensor connected to calli (arrow with the letter x) and oSE (arrow with the letter y), (I) tissue differentiation, reserve parenchyma cells (spherical and birefringent amyloplast) and procambium; scutellar somatic embryos (scSEs), (J) suspended in medium, (K) cross-section (toluidine blue, 200×), (L) scSE with procambium and a few vascular cells; coleoptilar somatic embryos (colSEs), (M) suspended in medium, cross-section (toluidine blue, 200×) high histodifferentiation in the region near the embryo, along the scutellum formed by reserve parenchyma cells and the axis defined by the meristem of the apex and root, (N) detail of a coleoptile and the apical and radicular meristem, (O) presence of the coleptile, polarity and tissue differentiation under light exposure. Am, amyloplast; col, coleoptile; e, embryo; EC, embryogenic cells; esc, scutellum; EZ, embryogenic zone; FM, fundamental meristem; MZ, meristematic zone; pa, procambial axis; Pa, parenchyma; PC, procambium; PD, protoderm; r, radicle; Rep, reserve parenchyma; S, suspensor.

Of the induced cultures, 73% formed yellow calli, 30% brown calli, and 50% suspended cells. Oblong somatic embryos (oSEs) (Fig. 1G) and scutellar somatic embryos (scSEs) (Fig. 1J) appeared earlier than expected in 6.25% of the cultures.

### Embryogenic proliferation

Proliferation of the nine morphogenetic products was, on average, similar among the three 2,4-D-containing treatments, but in all cases significantly greater than in the treatment without auxin (Table 1). Specifically, both the proliferation of yellow calli on the explant and suspended cells were significantly greater in the three 2,4-D treatments than in the treatment without auxin. A similar trend was observed for the proliferation of yellow calli suspended in the medium (Table 1). By contrast, the number of brown calli on the explant was significantly greater in medium without 2,4-D than in the other three treatments, in which the results were similar. Likewise, brown calli and scSEs suspended in the medium were only recorded in the treatment without auxin, albeit at low frequency (Table 1), whereas oSEs, both on the explant and suspended, were scarce in the three auxin treatments and completely absent in the treatment without auxin (Table 1). Thus, yellow calli were invariably associated with the presence of oSEs in the auxin treatments, and brown calli with scSEs in the auxin-free treatment. Based on the dominant calli developed in each treatment, four embryogenic lines were selected: brown calli in the treatment without auxin (BC0) and yellow calli in the three treatments with increasing auxin concentrations (YC1, YC2, and YC3) (Table 1; Table S1A).

**Table 1:** Percentage of cultures characterized by the proliferation of morphogenetic products of *Typha domingensis*.

Variable dependent	Embriogenic line			
	BC0	YC1	YC2	YC3
Yellow callus^e^	8.3 ± 7.9^b^	75.8 ± 11.2^a^	86.1 ± 8.3^a^	75.0 ± 12.5^a^
Brown callus^e^	86.1 ± 8.6^a^	9.1 ± 8.3^b^	11.1 ± 8.5^b^	25.0 ± 13.0^b^
oSE^e^	0.0	8.31 ± 4.3	16.7 ± 7.7	13.9 ± 7.6
scSE^e^	0.2 ± 3.7	0.0	0.1 ± 2.8	0.4 ± 8.8
Yellow callus^m^	16.7 ± 6.5	45.5 ± 13.9	33.3 ± 12.9	27.8 ± 10.7
Brown callus^m^	8.3 ± 8.3	0.0	0.0	0.0
oSE^m^	0.0	16.7 ± 16.9	13.9 ± 5.9	5.6 ± 5.6
scSE^m^	8.3 ± 5.9	0.0	0.0	0.0
Suspended cells	8.3 ± 8.3^b^	72.7 ± 13.0^a^	66.7 ± 14.2^a^	75.0 ± 13.0^a^
Total average	15.7 ± 3.2	26.9 ± 4.3	25.0 ± 3.9	26.2 ± 4.0

**Notes:**

Embryogenic lines: BC0, 0 mg L^−1^ 2,4-D; YC1, 0.5 mg L^−1^ 2,4-D; YC2, 1 mg L^−1^ 2,4-D; YC3, 2 mg L^−1^ 2,4-D.

e, adhered to explant; m, suspended in the culture medium; SE, somatic embryo.

Different letters (a, b) indicate significant differences (*p* < 0.01) by morphogenetic product.

### Maturation of somatic embryos

The embryogenic lines differed in their responses to light (Table 2), but significant differences were limited to yellow calli and suspended cells, with both yielding higher averages when cultivated in the dark. For oSEs on the explant, the average was almost twofold higher in dark-than in light-exposed cultures. The only absent embryogenic products were scSEs under conditions of light exposure and scSEs on the explant under conditions of dark exposure (Table 2).

**Table 2:** Percentages of cultures with morphogenetics products of *Typha domingensis* in the phase of embryogenic maturation.

Variable dependent	% of culture by treatment							
	BC0L	BC0D	YC1L	YC1D	YC2L	YC2D	YC3L	YC3D
Yellow callus^e^	0.0	0.0	33.3 ± 21^b^	66.7 ± 21^a^^b^	26.7 ± 12.5^b^	60.0 ± 24.5^a^^b^	66.7 ± 17.2^a^^b^	93.3 ± 6.7^a^
Brown callus^e^	93.3 ± 6.7^a^	86.7 ± 8.2^a^	40.0 ± 19.4^a^^b^	20.0 ± 20.0^b^	40.0 ± 24.5^a^^b^	46.7 ± 22.6^a^^b^	0.0	0.0
oSE^e^	0.0	0.0	0.0	20.0 ± 13.3	6.7 ± 6.7	13.3 ± 13.3	16.7 ± 14.9	6.7 ± 6.7
scSE^e^	0.0	0.0	0.0	0.0	6.7 ± 6.7^+^	0.0	33.3 ± 21.0^+^	0.0
Yellow callus^m^	0.0	0.0	20.0 ± 20.0^b^	60.0 ± 16.3^a^^b^	60.0 ± 24.5^a^^b^	26.7^a^ ± 16.3^b^	75.0 ± 22.3^a^	73.319.4^a^
Brown callus^m^	33.3 ± 21	6.7 ± 6.7	0.0	13.3 ± 13.3	0.0	0.0	41.7 ± 22.3	0.0
oSE^m^	0.0	0.0	20.0 ± 20.0	20.0 ± 20.0	6.7 ± 6.7	0.0	25.0 ± 22.3	0.0
scSE^m^	0.0	0.0	0.0	0.0	0.0	0.0	0.0	13.3 ± 8.1^a^
Suspended cells	0.0	0.0	60.0 ± 24.5^b^	100.0^a^	80.0 ± 20.0^a^^b^	100.0^a^	100.0^a^	100.0^a^
Total average	14.0 ± 5.0^dc^	10.4 ± 4.2^d^	19.2 ± 5.6^b^^c^^d^	33.3 ± 6.5^a^^b^	25.2 ± 6.9^a^^b^^c^^d^	27.4 ± 6.4^a^^b^^c^	39.8 ± 6.7^a^	31.9 ± 6.5^a^^b^

	% of culture by embriogenic line							
	BC0		YC1		YC2		YC3	
Yellow callus^e^	0.0		50.0 ± 15.1^b^		43.3 ± 15.1^b^		81.5 ± 9.7^a^	
Brown callus^e^	90.0 ± 5.0^a^		29.9 ± 13.6^b^		43.3 ± 15.7^b^		0.0	
oSE^e^	0.0		10.0 ± 7.1		10.0 ± 7.1		11.1 ± 7.4	
scSE^e^	0.0		0.0		3.3 ± 3.3		14.8 ± 10.7	
Yellow callus^m^	0.0		40.0 ± 13.9^a^		43.3 ± 14.94^a^		74.0 ± 14.3^a^	
Brown callus^m^	20.0		6.7		0		18.5	
oSE^m^	0		20.0 ± 13.3		3.3 ± 3.3		11.1 ± 10.5	
scSE^m^	0.0		0.0		0.0		7.4 ± 3.7^a^	
Suspended cells	0.0		80.0 ± 13.3^a^		90.0 ± 10.0^a^		100.0 ± 11.9^a^	
Total average	12.2 ± 4.0^b^		26.3 ± 4.3^a^		26.3 ± 4.4^a^		35.4 ± 4.4^a^	

	% of culture by ambient							
	Light				Dark			
Yellow calluse	29.82 ± 12.1^b^				55.00 ± 15.4^a^			
Brown callus^e^	45.6 ± 10.6				38.3 ± 10.3			
oSE^e^	5.2 ± 3.6				10.0 ± 4.9			
scSE^e^	8.8 ± 5.5^a^				0.0			
Yellow callus^m^	36.8 ± 11.0				40.0 ± 9.6			
Brown callus^m^	17.5 ± 8.0				5.0 ± 3.6			
oSE^m^	12.3 ± 7.1				5.0 ± 5.0			
scSE^m^	0.0				3.3 ± 2.3^a^			
Suspended cells	57.89 ± 11.3^b^				75.00 ± 9.9^a^			
Total average	23.78 ± 3.0				25.74 ± 3.0			

**Notes:**

e, adhered to explant; m, suspended in the culture medium; SE, somatic embryo.

Embryogenic lines: BC0, 0 mg L^−1^ 2,4-D; YC1, 0.5 mg L^−1^ 2,4-D; YC2, 1 mg L^−1^ 2,4-D; YC3, 2 mg L^−1^ 2,4-D; L, Light; D, Dark.

Averages with same literals were not significantly different.

Different letters indicate significant differences (*p* < 0.01) by morphogenetic product.

Based on the average percentages, the levels of the nine embryogenic products that developed were significantly greater in YC1–YC3 than in BC0. As expected, yellow calli were present in significantly higher amounts in the three respective embryogenic lines than in BC0. Among the three YC lines, the average was higher in YC3 than in YC1 and YC2, in which the results were similar (Table 2). Likewise, oSEs, both on the explant and in suspension, and suspended cells reached higher levels in the YC lines than in the BC0 line, but the differences between YC1, YC2, and YC3 were not significant (Table 2). By contrast, brown calli on the explant and in suspension were more abundant in the BC0 line than in the three YC lines. There was no new growth of yellow calli and oSEs in the BC0 line, whereas a few brown calli were registered in all three YC lines (Table 2). Few scSEs were observed in YC3, either on the explant or suspended in the medium (Table 2).

Overall, most of the significant differences and tendencies were observed among the embryogenic lines rather than in the responses to light, as the average of the nine products was significantly higher in the YC3 line under light conditions and scSE growth was recorded only in YC3, both in light- and dark-exposed cultures (Table 2; Table S1B).

### Histological descriptions

The calli of *T. domingensis* produced embryogenic cells and underwent early and late embryogenesis (Fig. 1). In nodular yellow calli (Fig. 1A), zones of high mitotic activity, comprised of small, isodiametric cells with prominent nuclei, were seen (Fig. 1B) together with zones indicating embryogenic adeptness (Fig. 1C). Early and late embryogenesis were present in all three culture phases (Fig. 1). Pro-embryogenesis was associated with the presence of nodular yellow calli through the formation of induced pro-embryogenic masses (PEMs). The globular somatic embryos (gSEs) that originated in the PEMs were characterized by radial development, differentiation of the three primary meristematic tissues (Fig. 1E), and the presence of the three fundamental meristems and the suspensor (Fig. 1F). A reduction in suspensor mass was observed in the embryogenic stages that followed. The oSEs featured parenchyma with abundant amyloplasts (Fig. 1I). Elongation of the gSEs originated from the oSEs (Fig. 1G). The embryogenic stages that followed were the scSEs (Fig. 1J) and coleoptilar somatic embryos (colSEs) (Fig. 1M), both with vascular cells, reserve parenchyma, and a defined axis. These mature somatic embryos (colSEs) were identified by the presence of the coleoptile, polarity, and tissue differentiation under light exposure (Fig. 1O). Late embryogenic stages could also be identified, despite the abundance of embryos with aberrant morphologies (e.g., fused, doubled over the axis, with overexpression or suppression of structural components).

**Figure 2:**
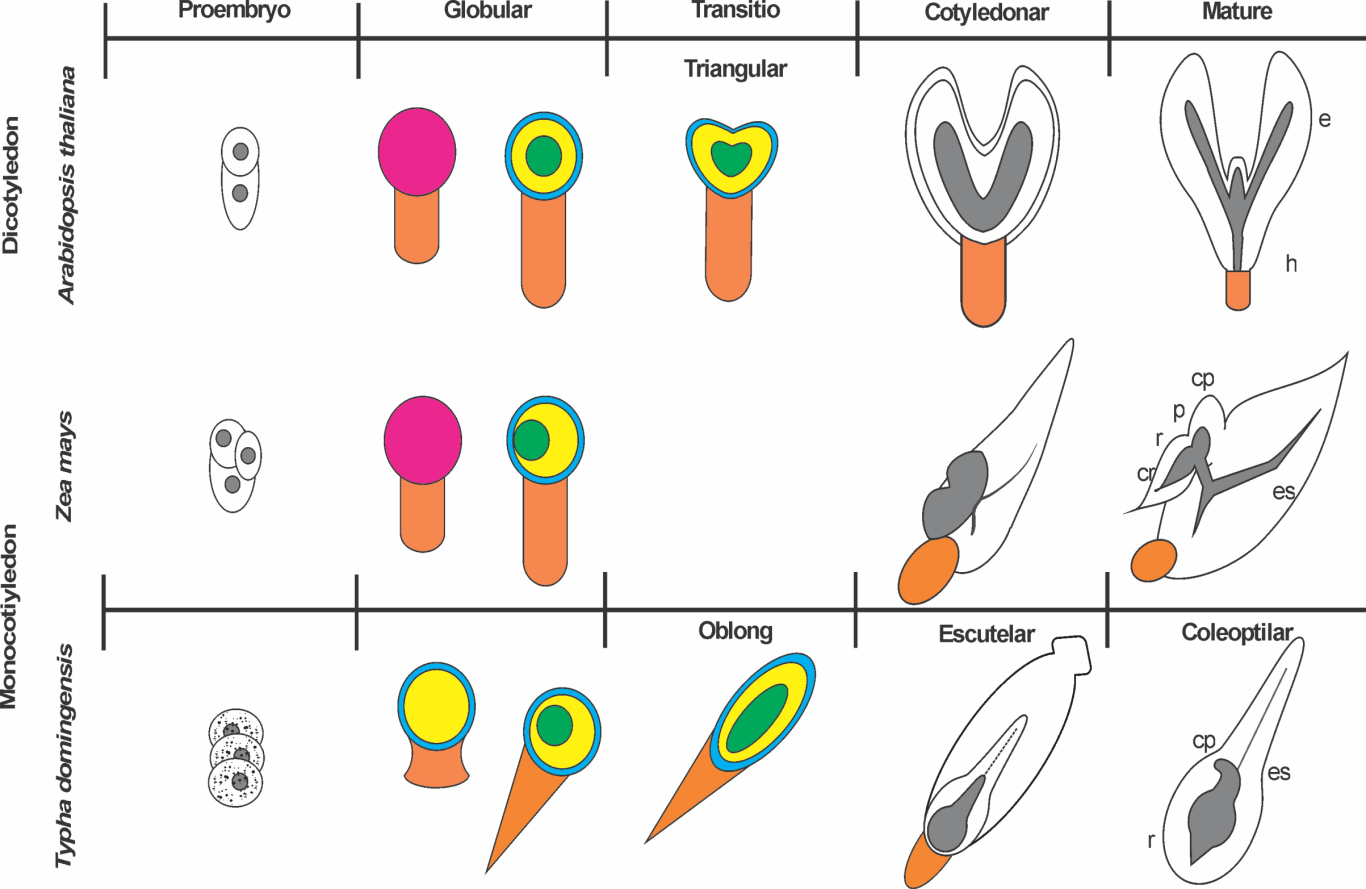
Histogenic model of the somatic embryogenesis of *Typha domingensis* compared with two model species: the dicotyledon *Arabidopsis thaliana* and the monocotyledon *Zea mays*. The illustrations are not to scale. cp, coleoptile; cr, coleorhiza; e, epicotyl; h, hypocotyl; p, plumule; r, radicle. Colors: yellow, fundamental tissue; green, procambium; blue, protoderm; orange, suspender; pink, zygote.

The cellular–histogenic differentiation enabled the creation of a roadmap describing the somatic embryogenesis of *T. domingensis* (Fig. 2), in which the sequence and degree of maturity of the SEs generated by the embryogenic lines of *T. domingensis* could be established based on the morpho-histological information obtained (Fig. S1).

## Discussion

Somatic embryogenesis of *T. domingensis* consisted of the early and late stages of induction, proliferation, and embryogenic maturation. Nevertheless, the process was asynchronous, because colSEs coexisted with embryos in the early stages of development. Nonetheless, the culture conditions and morphogenetic pathway in the embryogenic callus lines, which culminated in the formation of SEs derived from germinated seeds, represent a new route for the sustainable supply of cattail germplasm for conservation, repopulation, or industrialization. However, improvement of early embryogenic expression, using different auxins and doses thereof, balanced combinations of cytokinins, and coadjuvants in the culture medium, is still necessary, as is the evaluation of other explants (Gutiérrez-Mora et al., 2012; Abiri et al., 2017). Explant selection is controversial because the embryogenic response depends on complex interactions between the culture conditions and the physiological state of the explant at the time of cultivation. In this study, the selection of germinated seeds of *T. domingensis* as the explant was appropriate, given that at low auxin concentrations and in phytoregulator-free medium, induction of the embryogenic competence of the cells in the caulinar base of the germinated seedlings as well as the potential of this structure in the production of embryogenic calli were demonstrated. In other studies, germinated seeds of *Typha angustifolia* and inflorescences of *Phragmites australis* yielded embryogenic calli at lower concentrations (0.5 mg L^−1^) of 2,4-D (Lauzer, Dallaire & Vincent, 2000; Rogers, 2003; Burris et al., 2009). Likewise, explants of zygotic embryos of *Oryza sativa Indica* gave rise to the formation of embryogenic yellow calli with a minimum amount of necrotic material in MS medium containing 1.5 mg L^−1^ 2,4-D (Meneses et al., 2005). Yellow calli also developed at higher 2,4-D concentrations in other species and using other explants (Verdeil et al., 2001; Burris et al., 2009; Vega et al., 2009). In this study, the time spent in the explant culture during the inductive phase was important to embryo development, as the response was greater than expected. This can be explained by the redox effect of the added ascorbic acid, which enhances the embryogenic process (Dan, 2008), and/or by the ample fluctuation in the quality of the SEs and their rapid development. The low frequency of *Typha* SE formation was similar to that described for the *Indica* subspecies of rice (Indoliya et al., 2016), as well as two species of the genus *Phalaenopsis*, although the latter may have been due to the administration of 70% and 90% oxidation over long periods of light exposure (Gow, Chen & Chang, 2009).

The morpho-histological changes during the four stages of embryogenic differentiation provide insights into the mechanism of somatic embryogenesis in *Typha* and other unconventional aquatic monocotyledons, for which limited information has thus far been available. For example, in embryogenic line YC3, light exposure during the maturation phase promoted the growth of scSEs, which in turn influenced the formation of colSEs without causing necrotic damage.

In addition to the morphogenetic evidence, the histological study showed that the cellular organization and embryogenic differentiation of *T. domingensis* resembled that of other aquatic monocotyledons, such as *Panicum virgatum* (Burris et al., 2009), *O. sativa* (Vega et al., 2009; Bevitori et al., 2014), and *P. australis* (Máthé et al., 2000), as well as terrestrial monocotyledons such as *Cocos nucifera* and *Musa* spp. (Saenz et al., 2006; Strosse et al., 2006). In *T. domingensis*, transdifferentiation of meristematic cells to embryogenic cells was influenced by the addition of 2,4-D, because the callogenic activity of this auxin in light-exposed cultures led to maturation of the resulting embryos. A previous study demonstrated that the simultaneous presence of calli and early SEs in explants undergoing somatic embryogenesis coincided with the expression of a number of markers of zygotic embryos (Horstman, Bemer & Boutilier, 2017). The meristematic and embryogenic cells of the *T. domingensis* callus evolved to form nodules of meristematic tissue and PEMs. These histological characteristics also defined the pro-embryonic stage of *O. sativa* (Vega et al., 2009; Bevitori et al., 2014), *C. nucifera* (Saenz et al., 2006), and *Musa* spp. (Strosse et al., 2006). Early and late embryogenesis of *T. domingensis* resembled the sequential globular, oblong, scutellar, and coleoptilar stages of the zygotic embryos of other monocotyledons (Quiroz-Figueroa et al., 2006; Forestan, Meda & Varotto, 2010) and the developmental stages reported by Dodeman, Ducreux & Kreis (1997), Filonova, Bozhkov & Von Arnold (2000), Von Arnold et al. (2002), and Quiroz-Figueroa et al. (2006). Observation of the suspensor in *T. domingensis* was essential to determining the unicellular origin of the SE and its degree of development (Quiroz-Figueroa et al., 2006). The radial development plan of gSEs included three fundamental tissues typical of a spermatophyte (Winkelmann, 2016). The oSE of *T. domingensis* served as a transition between the gSE and scSE, as also reported for maize (Forestan, Meda & Varotto, 2010). The cotyledonary structure, with reserve parenchyma rich in amyloplasts, provided evidence of scutellar-type development by SEs of *T. domingensis*. In SEs of *O. sativa*, protoderm changes in the epidermis and the vascular bundle during the scutellar stage indicated the start of the next stage of development (Bevitori et al., 2014). In *T. domingensis*, the occasional vascular tissues seen in scSEs suggested their progression toward colSEs. The two embryogenic stages could be differentiated only by the coleoptile–radicle bipolarity of the latter. However, the two basic structures of a mature monocotyledon embryo, the coleorhiza and plumule, were not observed (Winkelmann, 2016; Forestan, Meda & Varotto, 2010). In *Zea mays* and *Arabidopsis thaliana*, stages of transitory development or of cellular expansion, rather than of differentiation, have been reported (Forestan, Meda & Varotto, 2010; Radoeva & Weijers, 2014). The high morphological variability of the SEs enabled the differentiation between normal embryos and abnormal or aberrant embryos resulting from a lack or overexpression of one or more structural elements, particularly during late stages (Hoenemann et al., 2010). Time-lapse tracking methods have been used to resolve problems associated with the coexistence of SEs in different stages of differentiation (Filonova, Bozhkov & Von Arnold, 2000), thus allowing the avoidance of side effects during the maturation process, such as the asynchronous activity of enzymes, signaling molecules, and the genetic pathways differentially expressed during somatic embryogenesis. In the case of the date palm, in the routine propagation of SEs, the production of aberrant embryos and shift to seedlings were prevented by applying a period of drying in polyethylene glycol (El Dawayati et al., 2012). However, the influence of light on the embryogenic process presents new challenges (Montgomery, 2016; Batista et al., 2018). The multistage monitoring of the cellular–histogenic differentiation of *T. domingensis* revealed both the sequence of events that make up somatic embryogenesis and the degree of maturity of the SEs. Also, these results should facilitate further studies on regeneration via somatic embryogenesis of emergent aquatic macrophytes. Morpho-histological characterization is based on the detection of cellular markers and the availability of cell lines with high regeneration potential (Rocha et al., 2018). Hence, the integration of histochemical, immunolocalization, flow cytometry, and in situ hybridization data will provide insights into cell competence during somatic embryogenesis (Rocha et al., 2016, 2018).

## Conclusions

The histological description of structurally complete colSEs and the presence of embryogenic products obtained in high abundance under light conditions during the maturation phase demonstrated the potential of somatic embryogenesis. The development of embryogenic lines, with and without auxin and under light or dark conditions, provides a basis for future research, including the synchronization of histological descriptions with the embryogenic stages of *Typha* spp. Moreover, the growth of abundant and diverse morphogenetic products opens the door to diverse lines of research into aquatic macrophytes and their phenotypic plasticity. For example, use of embryogenic lines produced with the highest concentration of auxin (e.g., YC3) offers a strategy for the regeneration of plants of the *Typha* genus, such as in the conservation and repopulation of wetlands.

## Supplemental Information

10.7717/peerj.5952/supp-1 Supplemental Information 1 Map of the embryogenic lines of *Typha domingensis* summarizing the morphological development of somatic embryos.

10.7717/peerj.5952/supp-2 Supplemental Information 2 Statistical summary tables. *e* = adhered to explant, *m* = suspended in the culture medium, SE = somatic embryo. *P* < 0.01** (very significant); *P* < 0.05* (significant); *P* < 0.1^+^ (marginally significant).

## Additional Information and Declarations

### Data Availability

The following information was supplied regarding data availability: Hernandez-Piedra, Guadalupe (2018): Somatic embryos of *Typha domingensis*.xlsx. figshare. Dataset. https://doi.org/10.6084/m9.figshare.7259207.

## References

[ref-1] Abiri R, Maziah M, Shaharuddin NA, Yusof ZNB, Atabaki N, Hanafi MM, Sahebi M, Azizi P, Kalhori N, Valdiani A (2017). Enhancing somatic embryogenesis of Malaysian rice cultivar MR219 using adjuvant materials in a high-efficiency protocol. International Journal of Environmental Science and Technology.

[ref-2] Batista DS, Felipe SHS, Silva TD, De Castro KM, Mamedes-Rodrigues TC, Miranda NA, Rios-Rios AM, Faria DV, Fortini EA, Chagas K, Torres-Silva G, Xavier A, Arencibia AD, Otoni WC (2018). Light quality in plant tissue culture: does it matter?. In Vitro Cellular & Developmental Biology - Plant.

[ref-3] Bevitori R, Popielarska-Konieczna M, Santos EM, Grossi-De-Sá ME, Petrofeza S (2014). Morpho-anatomical characterization of mature embryo-derived callus of rice (*Oryza sativa* L.) suitable for transformation. Protoplasma.

[ref-4] Burris JN, Mann DGJ, Joyce BL, Stewart CN (2009). An improved tissue culture system for embryogenic callus production and plant regeneration in switchgrass (*Panicum virgatum* L.). BioEnergy Research.

[ref-5] Dan Y (2008). Biological functions of antioxidants in plant transformation. In Vitro Cellular & Developmental Biology-Plant.

[ref-6] Devi K, Sharma M, Ahuja PS (2014). Direct somatic embryogenesis with high frequency plantlet regeneration and successive cormlet production in saffron (*Crocus sativus* L.). South African Journal of Botany.

[ref-7] Dodeman VL, Ducreux G, Kreis M (1997). Zygotic embryogenesis versus somatic embryogenesis. Journal of Experimental Botany.

[ref-8] El Dawayati MM, Abd El Bar OH, Zaid ZE, Zein El Din AF (2012). In vitro morpho-histological studies of newly developed embryos from abnormal malformed embryos of date palm cv. Gundila under desiccation effect of polyethelyne glycol treatments. Annals of Agricultural Sciences.

[ref-9] Elhiti M, Stasolla C, Wang A (2013). Molecular regulation of plant somatic embryogenesis. In Vitro Cellular & Developmental Biology-Plant.

[ref-10] Erwin KL (2009). Wetlands and global climate change: the role of wetland restoration in a changing world. Wetlands Ecology Management.

[ref-11] Fehér A (2015). Somatic embryogenesis–stress-induced remodeling of plant cell fate. Biochimica et Biophysica Acta (BBA)-Gene Regulatory Mechanisms.

[ref-12] Fehér A, Pasternak TP, Dudits D (2003). Transition of somatic plant cells to an embryogenic state. Plant Cell, Tissue and Organ Culture.

[ref-13] Filonova LH, Bozhkov PV, Von Arnold S (2000). Developmental pathway of somatic embryogenesis in *Picea abies* as revealed by time-lapse tracking. Journal of Experimental Botany.

[ref-14] Forestan C, Meda S, Varotto S (2010). ZmPIN1-mediated auxin transport is related to cellular differentiation during maize embryogenesis and endosperm development. Plant Physiology.

[ref-15] George EF, Hall MA, Klerk GJD, George EF, Hall MA, Klerk GJD (2008). Somatic embryogenesis. Plant Propagation by Tissue Culture.

[ref-16] Gow WP, Chen JT, Chang WC (2009). Effects of genotype, light regime, explant position and orientation on direct somatic embryogenesis from leaf explants of *Phalaenopsis* orchids. Acta Physiologia Plantarum.

[ref-17] Guillou C, Fillodeau A, Brulard E, Breton D, De Faria S, Dorothée V, Verdier D, Simon M, Ducos JP (2018). Indirect somatic embryogenesis of *Theobroma cacao* L. in liquid medium and improvement of embryo-to-plantlet conversion rate. In Vitro Cellular & Developmental Biology-Plant.

[ref-18] Gutiérrez-Mora A, González-Gutiérrez AG, Rodríguez-Garay B, Ascencio-Cabral A, Li-Wei L, Ken-Ichi S (2012). Plant somatic embryogenesis: some useful considerations. Embryogenesis.

[ref-19] Harrison MM, Tyler AC, Hellquist CE, Pagano T (2017). Phenolic content of invasive and non-invasive emergent wetland plants. Aquatic Botany.

[ref-20] He MX, Hu Q, Zhu Q, Pan K, Li Q (2015). The feasibility of using constructed wetlands plants to produce bioethanol. Environmental Progress & Sustainable Energy.

[ref-21] Hoenemann C, Richardt S, Krüger K, Zimmer AD, Hohe A, Rensing SA (2010). Large impact of the apoplast on somatic embryogenesis in *Cyclamen persicum* offers possibilities for improved developmental control in vitro. BMC Plant Biology.

[ref-22] Horstman A, Bemer M, Boutilier K (2017). A transcriptional view on somatic embryogenesis. Regeneration.

[ref-23] Indoliya Y, Tiwari P, Chauhan AS, Goel R, Shri M, Bag SK, Chakrabarty D (2016). Decoding regulatory landscape of somatic embryogenesis reveals differential regulatory networks between japonica and indica rice subspecies. Scientific Reports.

[ref-25] Klubicová K, Uvácková L, Danchenko M, Nemecek P, Skultéty L, Salaj J, Salaj T (2017). Insights into the early stage of *Pinus nigra* Arn. somatic embryogenesis using discovery proteomics. Journal Proteomics.

[ref-26] Lauzer D, Dallaire S, Vincent G (2000). In vitro propagation of reed grass by somatic embryogenesis. Plant Cell Tissue Organ Culture.

[ref-27] Liu D, Wu X, Chang J, Gu B, Min Y, Ge Y, Shi Y, Xue H, Peng Ch, Wu J (2012). Constructed wetlands as biofuel production systems. Nature Climate Change.

[ref-28] Lorenzen B, Brix H, Mc Kee KL, Mendelssohn IA, Miao S (2000). Seed germination of two everglade species, *Cladium jamaincense* and *Typha domingensis*. Aquatic Botany.

[ref-29] Mahdavi-Darvari F, Noor NM, Ismanizan I (2015). Epigenetic regulation and gene markers as signals of early somatic embryogenesis. Plant Cell, Tissue and Organ Culture.

[ref-30] Máthé C, Hamvas MM, Grigorszky I, Vasas G, Molnár E, Power B, Davey MR, Borbély G (2000). Plant regeneration from embryogenic cultures of *Phragmites australis* (Cav.) Trin. Ex steud. Plant Cell, Tissue and Organ Culture.

[ref-31] Meneses A, Flores D, Muñoz M, Arrieta G, Espinoza AM (2005). Effect of 2,4-D, hydric stress and light on indica rice (*Oryza sativa*) somatic embryogenesis. Revista de Biología Tropical.

[ref-32] Mikuła A, Pożoga M, Tomiczak K, Rybczyński JJ (2015). Somatic embryogenesis in ferns: a new experimental system. Plant Cell Reports.

[ref-33] Mitsch WJ, Bernal B, Nahlik AM, Mander Ü, Zhang L, Anderson CJ, Jørgensen SE, Brix H (2013). Wetlands, carbon and climate change. Landscape Ecology.

[ref-34] Montgomery BL (2016). Spatiotemporal phytochrome signaling during photomorphogenesis: from physiology to molecular mechanisms and back. Frontiers in Plant Science.

[ref-35] Mora-Olivo A, Villaseñor JL, Martínez M (2013). Las plantas vasculares acuáticas estrictas y su conservación en México. Acta Botanica Mexicana.

[ref-36] Murashige T, Skoog F (1962). A revised medium for rapid growth and bioassay with tobacco tissue culture. Physiologia Plantarum.

[ref-37] Oh MJ, Kim SW, Liu IR, Na HR, Choi HK (2008). High frequency plant regeneration from zygotic-embryo-derived embryogenic cell suspension cultures of watershield (*Brasenia schreberi*). Plant Biotechnology Reports.

[ref-38] Palomeque-De La Cruz MÁ, Galindo-Alcántara A, Escalona-Maurice MJ, Ruiz-Acosta SDC, Sánchez-Martínez AJ, Pérez-Sánchez E (2017). Analysis of land use change in an urban ecosystem in the drainage area of the Grijalva river, México. Revista Chapingo Serie Ciencias Forestales y del Ambiente.

[ref-39] Quiroz-Figueroa FR, Rojas-Herrera R, Galaz-Avalos RM, Loyola-Vargas VM (2006). Embryo production through somatic embryogenesis can be used to study cell differentiation in plants. Plant Cell, Tissue and Organ Culture.

[ref-40] Radoeva T, Weijers D (2014). A roadmap to embryo identity in plants. Trends in Plant Science.

[ref-41] Reed BM, Sarasan V, Kane M, Bunn E, Pence VC (2011). Biodiversity conservation and conservation biotechnology tools. In Vitro Cellular & Developmental Biology-Plant.

[ref-42] Reddy AR, Rasineni GK, Raghavendra AS (2010). The impact of global elevated CO_2_ concentration on photosynthesis and plant productivity. Current Science.

[ref-43] Rocha DI, Kurczyńska E, Potocka I, Steinmacher DA, Otoni W, Loyola-Vargas V, Ochoa-Alejo N (2016). Histology and histochemistry of somatic embryogenesis. Somatic Embryogenesis: Fundamental Aspects and Applications.

[ref-44] Rocha DI, Vieira LM, Koehler AD, Otoni WC, Loyola-Vargas V, Ochoa-Alejo N (2018). Cellular and morpho-histological foundations of in vitro plant regeneration. Plant Cell Culture Protocols. Methods in Molecular Biology.

[ref-45] Rogers SM (2003). Tissue culture and wetland establishment of the freshwater monocots *Carex*, *Juncus*, *Scirpus* and *Typha*. In Vitro Cellular & Developmental Biology-Plant.

[ref-46] Saenz L, Azpeitia A, Chuc-Armendariz B, Chan JL, Verdeil JL, Hocher V, Oropeza C (2006). Morphological and histological changes during somatic embryo formation from coconut plumule explants. In Vitro Cellular & Developmental Biology-Plant.

[ref-47] Sánchez-Chiang N, Jiménez VM (2010). Técnicas de conservación *in vitro* para el establecimiento de bancos de germoplasma en cultivos tropicales. Agronomía Mesoamericana.

[ref-48] Smertenko A, Bozhkov PV (2014). Somatic embryogenesis: life and death processes during apical-basal patterning. Journal of Experimental Botany.

[ref-50] Strosse H, Schoofs H, Panis B, Andre E, Reyniers K, Swennen R (2006). Development of embryogenic cell suspensions from shoot meristematic tissue in bananas and plantains (*Musa* spp.). Plant Science.

[ref-51] Thorp JH, Thoms MC, Delong MD (2006). The riverine ecosystem synthesis: biocomplexity in river networks across space and time. River Research and Applications.

[ref-52] Vega R, Vásquez N, Espinoza AM, Gatica AM, Valdez-Melara M (2009). Histology of somatic embryogenesis in rice (*Oryza sativa* cv. 5272). Revista de Biología Tropical.

[ref-53] Verdeil JL, Hocher V, Huet C, Grosdemange F, Escoute J, Ferriere N, Nicole M (2001). Ultrastructural changes in coconut calli associated with the acquisition of embryogenic competence. Annals of Botany.

[ref-54] Von Aderkas P, Teyssier C, Charpentier J, Gutmann M, Pâques L, Metté C, Ader K, Label P, Kong L, Lelu-Walter M (2015). Effect of light conditions on anatomical and biochemical aspects of somatic and zygotic embryos of hybrid larch (*Larix* × *marschlinsii*). Annals of Botany.

[ref-55] Von Arnold S, Sabala I, Bozhkov P, Dyachok J, Filonova L (2002). Developmental pathways of somatic embryogenesis. Plant Cell, Tissue and Organ Culture.

[ref-56] Wickramasuriya AM, Dunwell JM (2015). Global scale transcriptome analysis of Arabidopsis embryogenesis in vitro. BMC Genomics.

[ref-57] Winkelmann T, Germana MA, Lambardi M (2016). Somatic versus zygotic embryogenesis: learning from seeds. In Vitro Embryogenesis in Higher Plants.

[ref-58] Xu Z, Feng Z, Yang J, Zheng J, Zhang F (2013). Nowhere to invade: *Rumex crispus* and *Typha latifolia* projected to disappear under future climate scenarios. PLOS ONE.

