# Supplementary figures and images for: Morpho-histological development of the somatic embryos of Typha domingensis
Source: PeerJ. 2018 Nov 23;6:e5952. doi: 10.7717/peerj.5952 (PMC6254243; doi:10.7717/peerj.5952)

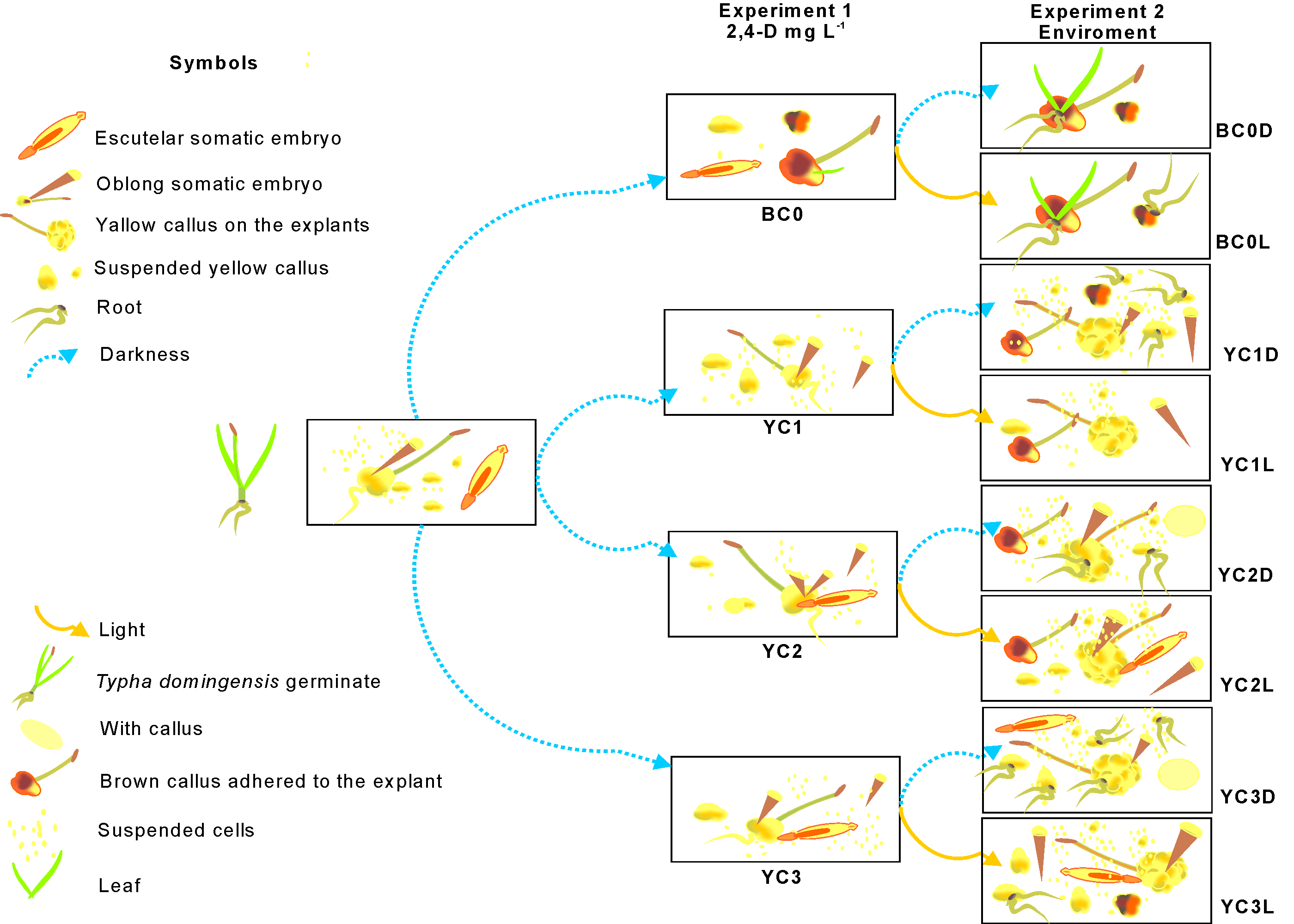

Supplement: Supplemental Information 1 [file peerj-06-5952-s001.tif]
