# Supplementary material for: Morpho-histological development of the somatic embryos of Typha domingensis
Source: PeerJ. 2018 Nov 23;6:e5952. doi: 10.7717/peerj.5952 (PMC6254243; doi:10.7717/peerj.5952)
Supplement: Supplemental Information 2 — e = adhered to explant, m = suspended in the culture medium, SE = somatic embryo. P < 0.01** (very significant); P < 0.05* (significant); P < 0.1+ (marginally significant). [file peerj-06-5952-s002.docx]

Table 1S. Statistical summary tables

Table 1SA. Embryogenic callus proliferation (experiment 1) summary table.

| Dependent variable | Statistical parameters | | | | | |
| --- | --- | --- | --- | --- | --- | --- |
|  | gl | SC | CM | H_(N=48)_ | F | P |
| Yellow callus ^e^ | 3 | 45599.8 | 15199.9 |  | 12.358 | 0.0001* |
| Brown callus ^e^ | 3 | 46649.8 | 15549.9 |  | 13.077 | 0.0001* |
| oSE ^e^ | 3 |  |  | 4.523 |  | 0.210 |
| scSE ^e^ | 3 |  |  | 3.849 |  | 0.278 |
| Yellow callus ^m^ | 3 | 4941.61 | 1647.2 |  | 1.076 | 0.369 |
| Brown callus ^m^ | 3 |  |  | 3.000 |  | 0.391 |
| oSE ^m^ | 3 |  |  | 3.562 |  | 0.312 |
| scSE ^m^ | 3 |  |  | 6.127 |  | 0.105 |
| Suspended cells | 3 | 36018.7 | 12006.2 |  | 6.441 | 0.001* |
| Total average | 3 | 8765.4 | 2921.8 |  | 1.803 | 0.145 |

e= adhered to explant, m= suspended in the culture medium, SE=somatic embryo.

p˂0.01** (very significant); p˂0.05* (significant); p˂0.1^+^ (marginally significant).

Table 1SB. Maturation of the four embryogenic lines (experiment 2) summary table.

| Dependent variable | Source of variation | Statistical parameters | | | | | |
| --- | --- | --- | --- | --- | --- | --- | --- |
|  |  | gl | SC | CM | H or U | F | P |
| Yellow callus ^e^ | Light/Dark | 1 | 5279.46 | 5279.46 |  | 4.046 | 0.053* |
|  | Embryogenic line | 3 | 31130.16 | 10376.72 |  | 7.953 | 0.000** |
|  | Interaction | 3 | 1885.71 | 628.57 |  | 0.481 | 0.697 |
|  | Treatment | 7 | 39441.6 | 5634.51 |  | 4.56985 | 0.001** |
| Brown callus ^e^ | Light/Dark | 1 | 242.44 | 242.44 |  | 0.187 | 0.667 |
|  | Embryogenic line | 3 | 40177.22 | 13392.41 |  | 10.379 | 0.000** |
|  | Interaction | 3 | 965.04 | 321.68 |  | 0.249 | 0.861 |
|  | Treatment | 7 | 41595.45 | 5942.21 |  | 4.60521 | 0.001** |
| oSE ^e^ | Light/Dark | 1 |  |  | 0.650 |  | 0.420 |
|  | Embryogenic line | 3 |  |  | 2.388 |  | 0.495 |
|  | Treatment |  |  |  | 5.497 |  | 0.599 |
| scSE ^e^ | Embryogenic line | 3 |  |  | 4.385 |  | 0.222 |
|  | Light/Dark | 1 |  |  | 3.328 |  | 0.068+ |
|  | Treatment |  |  |  | 13.495 |  | 0.069+ |
| Yellow callus ^m^ | Light/Dark | 1 | 15.15 | 15.15 |  | 0.010 | 0.919 |
|  | Embryogenic line | 3 | 26385.71 | 8795.24 |  | 5.963 | 0.002** |
|  | Interaction | 3 | 6766.67 | 2255.56 |  | 1.529 | 0.226 |
|  | Treatment | 7 | 33252.14 | 4750.31 |  | 3.22074 | 0.010** |
| Brown callus ^m^ | Light/Dark | 1 |  |  | 1.154 |  | 0.282 |
|  | Embryogenic line | 3 |  |  | 3.866 |  | 0.276 |
|  | Treatment |  |  |  | 10.100 |  | 0.183 |
| oSE ^m^ | Light/Dark | 1 |  |  | 1.140 |  | 0.285 |
|  | Embryogenic line | 3 |  |  | 2.245 |  | 0.523 |
|  | Treatment |  |  |  | 4.688 |  | 0.697 |
| scSE ^m^ | Light/Dark | 1 |  |  | 1.951 |  | 0.162 |
|  | Embryogenic line | 3 |  |  | 6.846 |  | 0.076+ |
|  | Treatment |  |  |  | 13.967 |  | 0.051+ |
| Suspended Cells | Light/Dark | 1 | 2181.8 | 2181.8 |  | 3.381 | 0.075+ |
|  | Embryogenic line | 3 | 61542.9 | 20514.3 |  | 31.797 | 0.000** |
|  | Interaction | 3 | 2685.7 | 895.2 |  | 1.387 | 0.264 |
|  | Treatment | 7 | 66666.7 | 9523.8 |  | 14.7619 | 0.000** |
| Total average | Treatment | 7 | 29871.4 | 4267.3 |  | 2.677 | 0.010** |
|  | Embryogenic line | 3 | 23726.6 | 7908.9 |  | 4.9640 | 0.002** |
|  | Ambient | 1 | 336.6 | 336.6 |  | 0.2038 | 0.651 |

e= adhered to explant, m= suspended in the culture medium, SE=somatic embryo.

p˂0.01** (very significant); p˂0.05* (significant); p˂0.1^+^ (marginally significant).
